# Different normalization strategies for microarray gene expression traits affect the heritability estimation

**DOI:** 10.1186/1753-6561-1-s1-s154

**Published:** 2007-12-18

**Authors:** Jun Ma, Zhaohui S Qin

**Affiliations:** 1 Department of Molecular Cellular and Developmental Biology, University of Michigan, 210 Washtenaw Avenue, LSI 6026, Ann Arbor, Michigan 48109-2216, USA; 2 Center for Statistical Genetics, Department of Biostatistics, University of Michigan, M4232 SPHII, 1420 Washington Heights, Ann Arbor, Michigan 48109-2029, USA

## Abstract

Several studies have been conducted to assess the influence of genetic variation on genome-wide gene expression profiles measured by the microarray technologies. Due to substantial noise in microarray-based experiments, it has long been recognized that proper normalization is a crucial step to ensure sensitive and reliable downstream analyses. This is especially true when large number of samples were collected and analyzed. In this study, we investigated the impact of different normalization strategies on genome wide linkage analyses, in particular, the estimation of heritability of gene expression traits. We used the Genetics Analysis Workshop 15 Problem 1 data. We found that there are significant differences in the estimated number of genes showing heritability when different normalization strategies were used. RMA (robust multiarray average) and dChip identify 45% and 13% more genes showing heritability than MAS 5.0, respectively. Our study also reveals that a large number of genes show strong "family effect" in their expression levels but no significant heritability. Analysis of their annotation indicates different types of genes were enriched in this group compared to genes showing strong heritability.

## Background

DNA microarray technologies provide a method to measure gene expression levels on a genomic scale. Recently, this technique has been applied in genetics studies to investigate the effects of genetic variants on gene expression levels [1-5]. This approach is referred to as genetical-genomics approach [6] in which gene expression levels were treated as quantitative traits. As expected, the accuracy and reliability of the expression measurements are essential and have significant impact on identifying loci that affect these quantitative traits. However, it has long been recognized that there is substantial intrinsic noise contained in microarray data. Removing systematic noise from raw microarray data is crucial for the downstream analyses. For Affymetrix GeneChip technology, which generated our data, there are two issues that need to be addressed. First, since a gene is represented by one or more probe sets, each contains series of perfect and mismatch probe pairs; a crucial step is to combine the intensity measures from multiple probes to produce a single value that best captures the expression level of the particular RNA transcript. Second, significant differences between chips have been observed due to various experimental artifacts; therefore it is important that a cross-chip normalization step is applied such that noise due to chip-specific experimental conditions can be removed to allow comparison across multiple chips.

An array of summarization and normalization strategies have been proposed to address these issues and are implemented in software such as MAS 5.0 [7], dChip [8], RMA (robust multiarray average) [9], among others. These methods are based on different statistical models, different summarization strategies, and different cross-chip normalization methods. As a consequence, the normalized gene expression profiles produced by these methods are quite different.

Results obtained from high level analyses such as detection of differentially expressed genes, clustering, and classification are often dependent on the summarization and normalization strategies used during the pre-processing step. Several studies have been conducted to compare the effects of various normalization strategies on high-level analyses [10-12]. More recently, the impact of various normalization strategies on genetical-genomics experiments conducted on recombinant inbred mouse strains have been evaluated and debated [13-15].

In this study, we used a novel design to study the consequences of different normalization strategies on gene expression trait heritability estimates. Our objectives are two-fold: first, heritability is an important measure in linkage studies as it is often used as a screening tool to select traits of interest. Whether the normalization step significantly influences the heritability measure is of great interest. Second, as pointed out by Chesler et al. [13], in microarray experiments, it is very difficult to determine "which method best approximates 'truth' in a situation in which truth is typically unknown", for example, determining differentially expressed genes. However, random noise alone is unlikely to produce gene expression pattern that show heritability in multiple multi-generation families, thus heritability measure in a large linkage study present a desirable setting to compare the sensitivity and specificity of various normalization methods. We hypothesize that noise associated with microarray experiments tends to eliminate or weaken the expression profile pattern of heritable gene such that heritability will be difficult to detect without proper normalization.

Here we use the Genetics Analysis Workshop 15 Problem 1 to test our hypothesis. We choose MAS 5.0, RMA and dChip because they are commonly used in analyzing Affymetrix GeneChip data. Other normalization strategies can be compared similarly. We used Bioconductor package [16] built on top of the R programming language to perform the analyses in this study.

## Methods

### Normalization strategy

Three normalization strategies were used in this study. MAS 5.0 is the updated version of the Affymetrix analysis algorithm. This method uses both the signals from perfect match and mismatch probes. The signal from a mismatch is calculated in such a way that it is never greater than the signal from a perfect match. dChip introduced a statistical model at the probe level to compute the so-called expression index. In such a model, both perfect match and mismatch probes are utilized. Using specific parameters to account for probe- and chip-specific effects, this model summarizies probe-level data and cross-chip normalization simultaneously. RMA introduces a new expression measure that is primarily based on perfect match probes. Mismatch probes were used to calculate background signal to non-specific binding. Specific distribution assumptions are made to make sure the transformed measurements are positive. A quantile normalization technique [10] is used to normalize expression levels from multiple chips.

### Data

Expression levels of genes in lymphoblastoid cells from 14 three-generation Centre d'Etude du Polymorphisme Humain Utah families (4 grandparents, 2 parents, 8 offsprings per sibship, for a total of approximately 14 individuals per family) were obtained using the Affymatrix Human Focus Arrays, which contain probes for 8792 transcripts. About 100 individuals' array hybridizations were performed in duplicate. In this study, only the first array of the duplicated individual was used. So gene expression data from 194 different individuals were analyzed.

After probe-level data were converted to expression levels and data log-transformed, a filtering step was applied to select differentially expressed genes for further analysis. This is necessary because genes that do not show much variation among individuals are non-informative in heritability estimation. We used two selection criteria for each gene, 1) the absolute difference (maximum-minimum) has to be greater than 1 and 2) the relative difference (maximum/minimum) has to be greater than 1.5. This variance filter is different from that used in Morley et al. [1] but it has been frequently used in microarray studies such as that of Tamayo et al. [17].

## Results

### Heritability

Following the filtering step, approximately 1750 genes were selected and subsequently processed by each of the three normalizing strategies. Heritability of these genes was then calculated by POLY software [18]. To correct for multiple testing, we computed the false-discovery rate (FDR) [19] using the *q*-value software [20]. The numbers of heritable genes after the three normalization strategies using two different FDR thresholds are summarized in Table 1.

**Table 1:** Number of heritable genes identified after using there different normalization methods

	RMA	dChip	MAS 5.0
No. selected genes for analysis	1737	1764	1785
No. heritable genes with FDR ≤ 0.05	994	791	696
No. heritable genes with FDR ≤ 0.01	757	584	520
Average no. false positives after permutation with *p*-value ≤ 0.05 (standard deviation)	24.4 (4.5)	43.8 (5.4)	39.1 (6.5)

**Figure 1:**
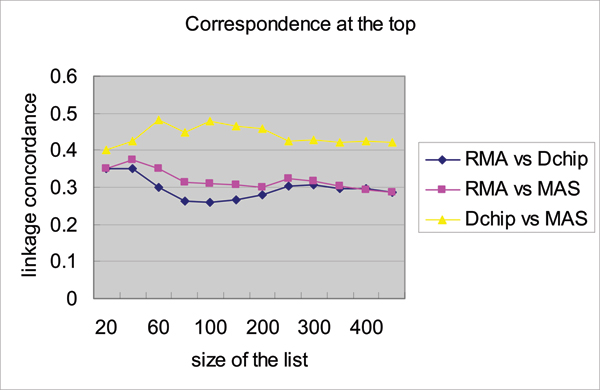
Correspondence analysis of three lists of heritable genes identified after using three different normalization strategies: MAS 5.0, dChip, and RMA.

Table 1 demonstrates the numbers of genes that pass the variance filter are roughly the same in each of the three categories; however the numbers of heritable genes identified by the three normalizing strategies differ. Using a FDR of 0.05, dChip and RMA identified 13.6% and 43.1% more heritable genes than MAS 5.0, respectively. When the FDR threshold is lowered to 0.01, the differences changed to 12.3% and 45.6%, respectively. Using RMA, 57.5% of genes that passed the variance filter display significant heritability pattern; their heritability estimates range from 0.11 to 0.97 with median of 0.26. To examine the overlaps of heritable genes detected under three different normalization strategies, we plotted the correspondence at the top (Figure 1) [21]. This plot indicates that the agreement between the three lists of heritable genes is low, ranging from 26% to 48%. We also measured the type I error rates of heritability estimates resulting from the three normalization strategies. From the 100 permutation tests performed, using nominal *p*-value of 0.05 as the cut-off, we found that the average proportions of genes incorrectly detected as heritable are 2.2%, 2.5%, and 1.4% for MAS 5.0, dChip, and RMA, respectively (Table 1). This indicates that these normalization strategies are conservative and RMA in fact displays the lowest type I error rate.

### Family-specific traits

**Figure 2:**
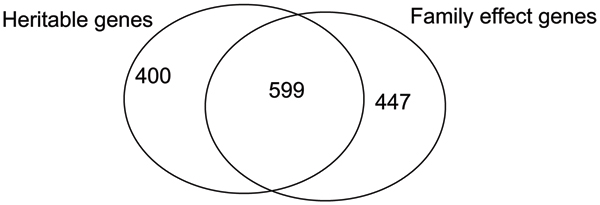
Venn diagram of genes that show heritability and/or family specific effect.

We also found that a large number of genes display strong family specific effects; that is, a majority of the overall variance in the expression levels comes from across families. We first used RMA to normalize expression levels and then used one-way ANOVA to identify genes showing family-specific expression patterns. Although the majority of these genes show heritability in their gene expression trait, about 40% fail to show significant heritability using nominal *p*-value of 0.05 as the cut-off. We speculate that the expression levels of these genes may be affected by non-genetics factors such as environment or population substructures. Venn diagrams on genes showing heritability and family-specific effects can be found in Figure 2.

### Comparison of annotations

To determine which functional categories are enriched among genes that show patterns of heritability and/or family-specific effects, we analyzed three lists of genes using GOstat [22], a software tool that calculates the statistical significance of the gene ontology (GO) term enrichment. The three lists are genes that show heritability and family-specific effects, genes that show heritability but no family specificity, and genes that show family-specific effects but no heritability. In this analysis, we only used expression levels obtained using RMA. Table 2 summarize the results. From this table, we found that the most notable functional category for genes that display both heritability and family-specific effect is apoptosis, a major type of program cell death. Apoptosis plays an important role in development, immune cell regulation, and response to cell damage. Another notable feature is that significant functional categories in these three groups differ, which indicates significant difference among these three groups of genes.

**Table 2:** Annotation comparison of genes that show heritability and/or family specific effect

Heritable and family effect	*p*-Value	Family effect non-heritable	*p*-Value	Heritable non-family effect	*p*-Value
Protein binding	3.71 × 10^-13^	Intracellular part	2.99 × 10^-24^	Protein binding	7.12 × 10^-19^
Intracellular part	4.83 × 10^-11^	Cytoplasm	8.73 × 10^-22^	Integral to plasma membrane	5.58 × 10^-9^
Apoptosis	4.83 × 10^-11^	Intracellular membrane bound organelle	5.14 × 10^-18^	Intrinsic to plasma membrane	5.68 × 10^-9^
Programmed cell death	4.83 × 10^-11^	Intracellular organelle	3.32 × 10^-17^	Receptor binding	6.99 × 10^-8^
Plasma membrane	1.66 × 10^-10^	Protein binding	2.37 × 10^-16^	Plasma membrane	6.99 × 10^-8^
Cell death	1.76 × 10^-10^	Mitochondria	7.5 × 10^-16^	Intracellular part	1.08 × 10^-7^
Organ development	1.76 × 10^-10^	Cytosol	4.11 × 10^-15^	Regulation of cell proliferation	7.81 × 10^-7^
Cell proliferation	1.76 × 10^-10^	Cell cycle	3.53 × 10^-13^	Intracellular organelle	2.12 × 10^-6^
Intracellular membrane-bound organelle	6.54 × 10^-10^	Organelle lumen	2.04 × 10^-11^	Cell communication	3.34 × 10^-6^
Cell part	6.54 × 10^-10^	Nucleus	6.51 × 10^-11^	Morphogenesis	4.51 × 10^-6^

## Discussion and conclusion

This study assesses the impact of different normalization strategies on heritability estimation in genetical-genomics studies. Using Genetics Analysis Workshop 15 Problem 1 and three different methods (RMA, dChip, and MAS 5.0) we found that normalization strategies used to summarize data across multiple chips significantly influences the identification of heritable genes. The percentage of overlap between heritable genes identified using different methods is low. Among the three methods tested, RMA reveals the greatest number of heritable genes, followed by dChip and MAS 5.0. Interestingly, RMA also yields the least number of false positives in the permutation test. It is known that RMA performs well in cross-chip normalization, which may explain its highest sensitivity and specificity among the three methods tested. However, more studies are needed before a general conclusion can be drawn. The permutation test also suggests that all three methods produce conservative results in terms of heritability estimates. We also found a large number of genes with expression patterns that show significant family-specific effect, but no significant heritability. Annotation analysis indicates that different functional categories are enriched among these genes relative to heritable genes, indicating that perhaps these genes are involved in different functional mechanisms.

Recent genome-wide linkage scans of the same data set have indicated that normalization methods play an important role in the linkage results. These and our studies suggest that close attention needs to be paid to the pre-processing steps because they significantly impact the downstream analyses.

## Competing interests

The author(s) declare that they have no competing interests.
